# Trend of measles-rubella vaccination coverage and impact on measles epidemiology in the Savannah Region, Ghana; 2018–2022: A secondary data analysis

**DOI:** 10.1016/j.vaccine.2024.02.024

**Published:** 2024-03-19

**Authors:** Michael Rockson Adjei, Amos Longsignikuu, Ibrahim Saeed Iddris, Thomas Nang Suuri, Byrite Asamoah, Michael Okoye, Janet Vanessa Baafi, Chrysantus Kubio, Sally-Ann Ohene, Martin Peter Grobusch

**Affiliations:** a Center of Tropical Medicine and Travel Medicine, Department of Infectious Diseases, Amsterdam University Medical Centers, Iocation AMC, University of Amsterdam, Amsterdam, the Netherlands; b World Health Organization, Country Office, Accra, Ghana; c Ghana Health Service, Regional Health Directorate, Savannah Region, Damongo, Ghana; d Synlab, Accra, Ghana; e Acacia Health Insurance, Accra, Ghana; f Ghana Health Service, District Health Directorate, Sunyani West, Odumase, Ghana; g Institute of Tropical Medicine, and German Center of Infectious Diseases (DZIF), University of Tuebingen, Tuebingen, Germany; h Institute of Infectious Diseases and Molecular Medicine, University of Cape Town, Cape Town, South Africa; i Centre de Recherches Médicales En Lambaréné (CERMEL), Lambaréné, Gabon; j Masanga Medical Research Unit, Masanga, Sierra Leone

**Keywords:** Vaccination coverage, Dropout rate, Measles outbreak, Measles-rubella vaccine, Ghana, Savannah Region

## Abstract

**Introduction:**

Ghana witnessed an outbreak of measles in 2022 following the COVID-19 pandemic, and Savannah Region was among the regions severely impacted. The objective of this study was to conduct trend analysis of measles case incidence and measles-rubella (MR) vaccination coverage in the Savannah Region to identify gaps and propose remedial actions to mitigate future outbreaks of vaccine preventable diseases (VPDs).

**Methods:**

Analysis of measles surveillance and measles-rubella vaccination data for 2018–2022 was conducted to assess relationship between immunization coverage and measles case incidence. Data were extracted from the District Health Information Management System (DHIMS) platform and loaded into Microsoft Excel 16.0 spreadsheet for analysis. Coverages for first (MR1) and second (MR2) doses of measles-rubella vaccination, dropout rates, and measles incidence (per 100,000) were calculated.

**Results:**

The coverage trend for both vaccine doses followed similar trajectories, increasing from 2018 to a peak in 2019, and declining sequentially thereafter to the lowest (for the study period) in 2022. Generally, MR1/MR2 dropout rate was high across all districts during the entire study period. The regional incidence of confirmed measles rose sharply from less than 1/1,000,000 in 2018–2021 to 94 in 2022. Wide variations in vaccination coverage and dropout rates were observed among the districts. There was moderate to fairly strong negative correlation between MR vaccination coverage and measles case incidence.

**Conclusions:**

The MR vaccination coverage in the Savannah Region declined probably due to pre-existing weaknesses in the immunization programme accentuated by impact of the COVID-19 pandemic. The lowered population immunity likely contributed to occurrence of the measles outbreak in 2022. Pragmatic actions are needed to catch-up on missed children, restore coverage to pre-pandemic levels, and strengthen the immunization programme as part of global efforts towards achieving the Immunization Agenda 2030 (IA2030) trajectory.

## 1 Introduction

Vaccination is a proven cost-effective public health intervention with significant impact on reduction of childhood morbidity and mortality globally [1]. Immunization has a higher return on investment than many other interventions like pre-school education, public infrastructure, community health workers, government bonds and cardiovascular disease research. At comparably low cost, vaccines can prevent disease and disabilities, providing protection that lasts a lifetime, saving millions of dollars in potential healthcare spending by households and the health system [1], [2].

The global under-five mortality rate declined by 59 per cent, from 93 deaths per 1,000 live births in 1990 to 38 in 2021 [3]. It is estimated that globally, vaccines prevent up to three million deaths [4] annually. Despite this considerable progress, improving child survival remains a matter of urgent concern. In 2021 alone, roughly 13,800 under-five deaths occurred every day, amounting to an intolerably high number of largely preventable childhood deaths [3], especially those occurring from vaccine preventable diseases (VPDs).

The COVID-19 pandemic impacted severely on all aspects of life including health services all over the globe [5]. In Ghana, a study by Kissi et al. (2022) from the Greater Accra Region documented a substantial disruption in the provision of immunization services as a result of the COVID-19 pandemic. Administration of the various antigens declined [6].

Measles is fatal, with mortality rates of 2–15% among young children in LMICs. In 2018–2019, a global resurgence of measles occurred, affecting all WHO regions [7]. The emergence of COVID-19 disrupted the global fight against measles by impairing immunization programmes. This resulted in deceleration of the routine immunization drive due to diversion of resources towards the pandemic response with about 23 million children missing out on all basic childhood vaccines including measles-containing vaccines [8]. According to the World Health Organization, Africa is encountering a surge in outbreaks of VPDs since the last couple of years. About 17,500 cases of measles were recorded in the African Region between January and March 2022, indicating a 400% increase compared with the same period in 2021 [9].

Ghana has experienced a drastic decline in measles cases following the introduction of measles vaccine in 1978, as part of the World Health Organization (WHO)’s Expanded Programme of Immunizations (EPI); which since then has emerged to become the Essential Programme of Immunization). Between 2018 and 2022, Ghana maintained first dose measle-rubella (MR1) administrative coverage of at least 95%, and second dose (MR2) coverage of at least 80% [10]. According to the Ghana Demographic and Health Survey conducted in 2022, 87% of children aged 12–23 months had received MR1 while 73% aged 24–35 months had been vaccinated for MR2 [11].

The country has a well-functioning measles surveillance system embedded within the Integrated Disease Surveillance and Response (IDSR) system, and between 2019 and 2021 all 261 districts reported and investigated at least one suspected measles case for each year [Ghana Health Service, 2021 Annual Report, unpublished]. Despite the remarkable efforts, the country’s quest to eliminate measles by 2020 was not realized due to confirmation of 88 cases in 2020; 1,274 cases in 2021; and 395 cases in 2022 [12], [13]. With measles being a tracer disease, outbreaks correlate with decline in population immunity from reasons including suboptimal immunization programme performance [14].

The Savannah Region is emerging as epicenter for VPD outbreaks having confirmed a total of 87 cases (13.3/100,000 population) in the yellow fever epidemic that occurred in Ghana in 2021 (Savannah Regional Health Directorate, Annual Report 2021, unpublished). In 2022, it was among regions that were severely impacted by the measles outbreak. The objective of this study was to conduct trend analysis of measles case incidence and measles-rubella vaccination coverage in the region to identify gaps and propose remedial actions to mitigate future outbreaks.

## 2 Methods

### 2.1 Study design

Secondary analysis was conducted on measles surveillance data and measles-rubella vaccination data for 2018–2022.

### 2.2 Study setting

The Savannah Region was carved out from the Northern Region in 2019. It is bordered in the north by the Upper West and North East regions, in the west by Côte d'Ivoire, in the south by the Bono and Bono East regions, and in the east by Northern regions. It is the largest region in Ghana by land size and occupies an area of 35,862km^2^, with a total population of 649,627 comprising 50.2% of males. The region is arid, with one rainy season occurring typically annually between July and October. The majority of the inhabitants is peasant farmers pursuing grain farming and livestock rearing; most of the livestock farmers are undocumented nomads from neighbouring countries [15].

**Fig. 1:**
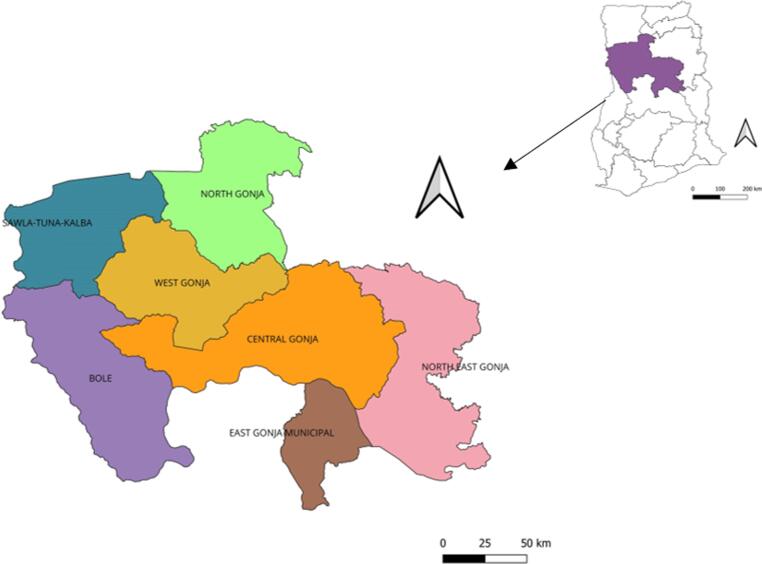
Map of study sites, Savannah Region, Ghana.

There are seven administrative districts (Fig. 1) and a total of 179 health care facilities comprising five hospitals, three polyclinics, 23 health centres, and 148 community-based health planning and services (CHPS) compounds. Most (>50%) of the communities are hard-to-reach and located more than five km from the nearest health facility. Immunization services are provided through a fixed strategy (facility-based vaccination sessions), and mobile strategy (community-to-community vaccination sessions). There is a generally poor road network, and access to communities off the main road network is worsened during the rainy season (July to October) due to springing-up of water bodies leading to floodings, which affects health service delivery.

### 2.3 Data collection

The regional population and population for surviving infants (children living beyond neonatal period) for 2018–2022 were extracted from District Health Information Management System (DHIMS). Similarly, the number of children receiving MR1 and MR2; and the number of suspected and confirmed measles cases were extracted from the database.

DHIMS is free open-source software (https://www.dhis2.org) developed from the district health information system (DHIS). It was first introduced in Ghana in the year 2007 and was primarily used for collecting and reporting health data. Data is collected at the health facility level using standard registers, collated onto summary forms, and entered into the DHIMS platform weekly or monthly. The interphase is password protected and accessible only by authorized healthcare managers. Data is extracted and shared spontaneously or upon request from health partners including WHO and UNICEF.

Data was collected by district into a Microsoft Excel 16.0, and the number of children vaccinated was verified from the source, checking monthly reports for completeness and consistency.

### 2.4 Data analysis

Data was analyzed in Microsoft Excel 16.0. Measles coverage was calculated by dividing the number of children vaccinated for the respective doses (MR1 and MR2) with the total population of surviving infants, multiplied by 100. The dropout rate was estimated by dividing the difference between the number of children vaccinated for MR1 and MR2 with the number vaccinated for first dose, multiplied by 100 [16]. The measles incidence (/1,000,000) was estimated by dividing the number of cases with the total population, multiplied by 1,000,000. This was done separately for suspected cases (MSC) and confirmed cases (MCC). Correlation between measles incidence and MR vaccination coverage was assessed at the regional level.

The computations were repeated for each year in the period under study (2018–2022). Results were displayed by place and time using text, tables, and charts.

### 2.5 Ethical clearance

Ethical clearance was sought from the Ghana Health Service Ethics Review Committee (ID number: GHS-ERC 002/10/23). Administrative permission was obtained from the Savannah Regional Health Directorate for collection and use of the institutional data. Patient names and/or identifiers were anonymized to ensure privacy and confidentiality. The data were stored on a computer protected with a password and used specifically for the study.

## 3 Results

Average data completeness and consistency were 100%, and 92% respectively. Generally, the trend of coverage for both vaccine doses followed similar trajectories, increasing from 2018 to a peak in 2019, and declining sequentially thereafter (Fig. 2A, Fig. 2B, Fig. 2C, Fig. 2D, Fig. 2E, Fig. 2F, Fig. 2G, Fig. 2H). For MR1, the regional coverage ranged from 109.5% in 2019 to 88% in 2022, respectively. Similarly, the coverage of MR2 ranged between 98.9% in 2019 and 72.9% in 2022 (Fig. 2A). However, doses administered for MR1 increased from 23,531 in 2018 to 25,319 in 2021 before dropping to 24,138 in 2022. For MR2, doses administered increased from 19,577 in 2018 to a peak of 21,372 in 2020, followed by a decline to 19,440 in 2022. There was no significant difference in the average number of vaccine doses administered annually in the pre-pandemic and pandemic periods (44,968 versus 43,906; p=0.8875).

**Fig. 2A:**
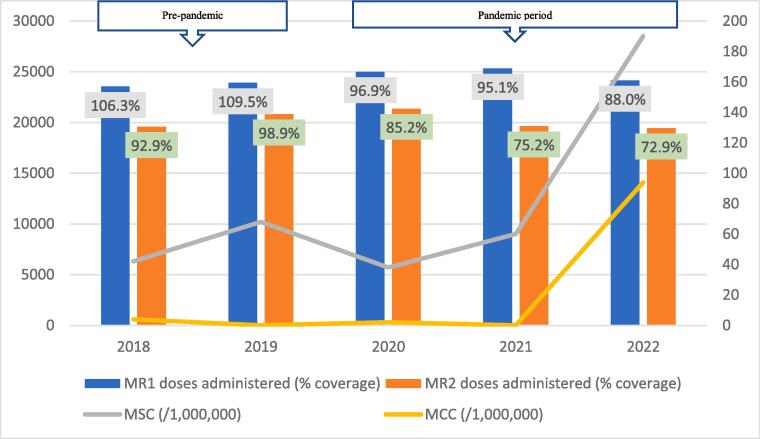
Measles-Rubella vaccination coverage and measles incidence, Savannah Region; 2018–2022.

**Fig. 2B:**
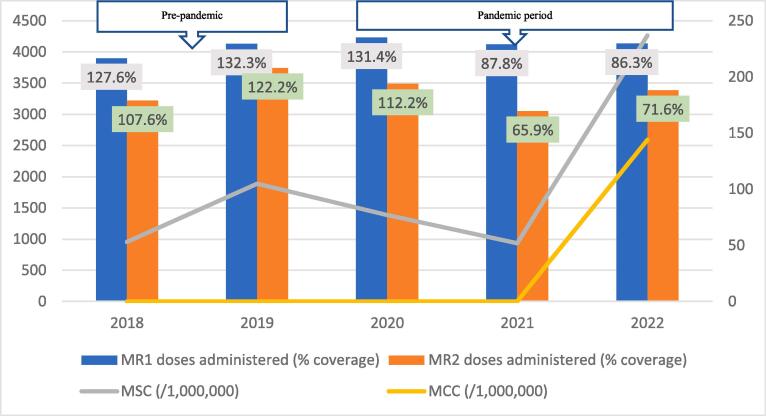
Measles-Rubella vaccination coverage and measles incidence, Bole District; 2018–2022.

**Fig. 2C:**
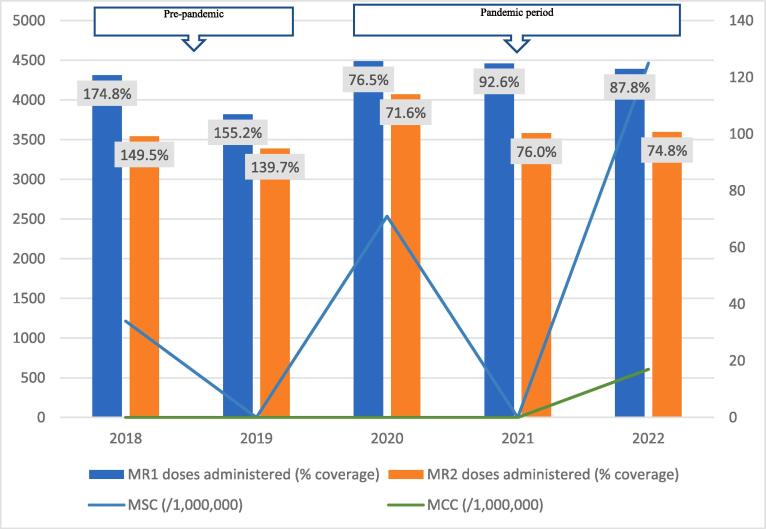
Measles-Rubella vaccination coverage and measles incidence, East Gonja District; 2018–2022.

**Fig. 2D:**
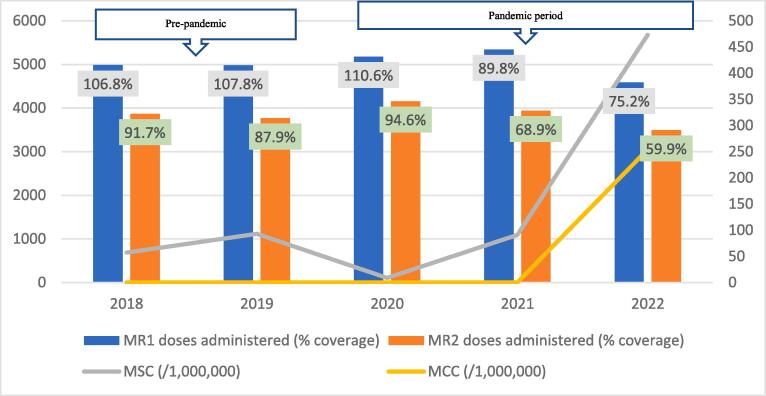
Measles-Rubella vaccination coverage and measles incidence, Central Gonja District; 2018–2022.

**Fig. 2E:**
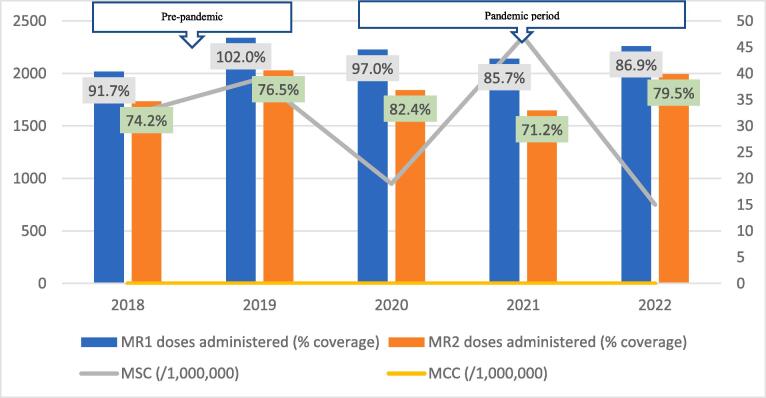
Measles-Rubella vaccination coverage and measles incidence, North Gonja District.

**Fig. 2F:**
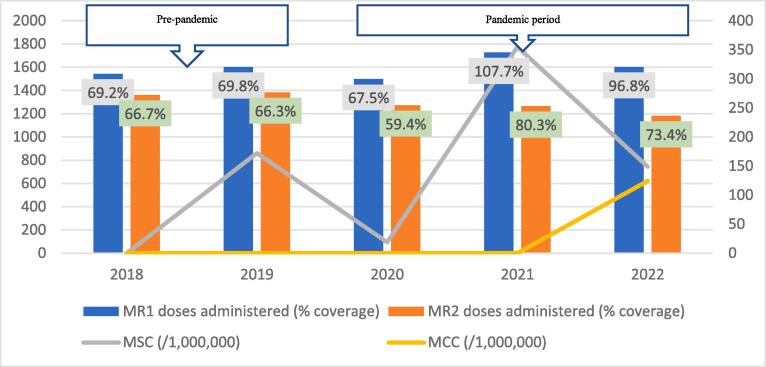
Measles-Rubella vaccination coverage and measles incidence, North East Gonja District; 2018–2022.

**Fig. 2G:**
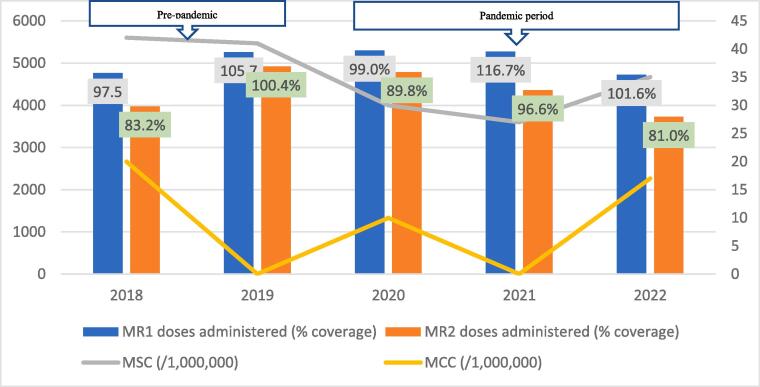
Measles-Rubella vaccination coverage and measles incidence, Sawla-Tuna-Kalba District; 2018–2022.

**Fig. 2H:**
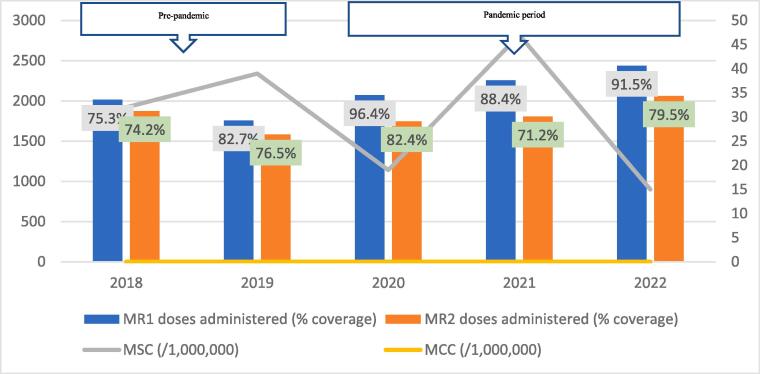
Measles-Rubella vaccination coverage and measles incidence, West Gonja District; 2018–2022.

The regional trend of vaccine uptake was largely reflected in all districts except in West Gonja where there was stepwise increase in doses administered during the years in the pandemic period (Fig. 2H). Generally, the trend in MR1/MR2 dropout was inversely related to the vaccination coverage with the lowest dropout rate being recorded in 2019 (12.9%) and the highest (22.4%) in 2021 (Table 1). All the districts recorded dropout rates of above 10% for at least three years of the 5-year study period (Table 1).

**Table 1:** Trend of MR1/MR2 dropout rate by district, Savannah Region; 2018–2022.

**District/REGION**	**Year**				
	**2018**	**2019**	**2020**	**2021**	**2022**
Bole	17.4	9.4	17.5	25.9	18.1
Central Gonja	22.4	24.4	19.7	26.3	23.8
East Gonja	17.9	11.4	9.2	19.7	18.1
North Gonja	14.1	13.3	17.4	23.1	11.8
North-East Gonja	11.8	13.6	15.1	26.8	26.3
Sawla-Tuna-Kalba	16.4	6.5	9.6	17.4	21.1
West Gonja	7	10	15.6	19.9	15.5
SAVANNAH	16.8	12.9	14.5	22.4	19.5

The regional incidence of confirmed measles was less than 1/1,000,000 for the first four years of the study period (2018–2021) but rose sharply to 94 in 2022; a period coinciding with the outbreak. This trend was reflected in all districts except North Gonja (Fig. 2E) and West Gonja (Fig. 2H) where no cases were confirmed for the entire study period. However, all the districts (except East Gonja; Fig. 2C) suspected and investigated at least one case in each year of the study period. There was moderate to fairly strong negative correlation between MR vaccination coverage and measles incidence although it was statistically insignificant (Table 2).

**Table 2:** Correlation coefficient (R) for MR coverage and measles incidence.

**Vaccination coverage**	**MSC (/1,000,000)**		**MCC (/1,000,000)**	
	**R**	**p-value**	**R**	**p-value**
MR1	−0.658	0.2273	−0.712	0.1773
MR2	−0.573	0.3125	−0.599	0.2858

## 4 Discussion

The study highlights the declining MR vaccination coverage and inequities in utilization among districts in Savannah Region, Ghana. Globally, health care services were disrupted by the COVID-19 pandemic resulting in rollback of the gains made in immunization over the years. In Ghana, capacity building of health workers, provision of guidelines and logistics by the Ministry of 10.13039/100018696Health of Ghana restored immunization service delivery within six months (as of July 2020) of the pandemic.

Despite the country’s intervention, immunization coverage in the Savannah Region continued to decline due to inherent geographical and programmatic challenges. The vast nature of the region, coupled with poor road network, inadequate health facilities, and migration patterns affected access and uptake of immunization services.

It is instructive to note that the inconsistencies between MR doses administered and coverages might largely be due to annual changes in the denominator (target population). Census remains a reliable source of population data and in Ghana, this is conducted every ten years [15]. Post census, annual target populations are projected from preceding years using the growth rate and other relevant parameters. Unreliable denominators – especially in situations where the target population is underestimated – could give spuriously high coverages (more than 100%) and healthcare workers may relax immunization defaulter catch-up measures. This phenomenon might have played out especially in Bole, Central Gonja, and East Gonja districts. Unrealistic immunization coverage is of much public health importance as low coverage, as both could lead to build up of un- or under-vaccinated population .

Although there was no significant difference between average number of vaccine doses administered annually in the pre-pandemic and pandemic periods, the low coverages observed in the latter presupposes that supplies were inadequate for the target population [17]. Generally, all districts recorded stagnation or decline in the yearly administered vaccine doses during the pandemic period, except West Gonja where sequential increases were observed. The location of the regional vaccine cold room in Damongo, the capital of West Gonja, conferred the advantage of being among the first districts to pick up stock of vaccine whenever supplies - albeit limited - were received from the national level.

The drop in MR1 and MR2 coverages by 19.6% and 26.3% between 2019 and 2022, respectively, is consistent with the findings of Bimpong et al (2021) [18] who reported a 10.5%-47% decline in uptake of various routine antigens due to the pandemic. The negative correlation coefficient observed by our study highlights the association between low measles vaccination coverage and risk of measles outbreak [8]. Although the coefficients were statistically insignificant, their clinical importance cannot be underestimated.

Ghana organizes MR supplemental immunization activities (SIAs) every three to four years to bridge immunity gap that might occur due to accumulation of un- and under-immunized population over time. The last SIA was conducted in 2018 [19], but the 2021/2022 edition had to be postponed due to the COVID-19 pandemic. In 2022, the country experienced stockout of measles-rubella vaccines due to insufficient funding of the immunization programme [20]. The delayed SIA coupled with vaccine stockouts interrupted the immunization coverage recovery efforts and might have contributed to the measles outbreak [16], [17].

The measles surveillance system in the region was sensitive and attained non-measles non-rubella febrile rash rate of more than 2/100,000 in each year of the study period despite low representativeness possibly due to gaps in human resource and laboratory capacity (including delays in receiving feedback), and resource availability [20]. North Gonja and West Gonja districts recorded no confirmed measles cases throughout the study period but achieved the benchmark for sensitivity in each year of the study period. The reason for the observation is unclear given that the two districts share similar socio-demographic and immunity profiles with those documenting confirmed cases.

Dropout rates between 0 and 10% are generally acceptable to accommodate inevitable reasons (such death) for discontinuation of vaccination schedule [21]. The high MR1/MR2 dropout rate was probably due to poor continuity of service utilization in the region, as was reflected in all the districts. According to Nyaku et al (2017), key factors for immunization dropout include low caregiver knowledge and absence of robust defaulter tracking systems [22]. While low caregiver knowledge may reflect inadequate access to health education, some caregivers do not return for subsequent doses due to forgetfulness or competing priorities despite having been informed meticulously by the health worker [23], [24]. Again, the seasonality of economic activities including farming causes many caregivers – especially nomads – to move out of the region in search of arable lands or an alternative livelihood [25] resulting in low continuity of immunization. The challenging terrain makes defaulter tracing resource-intensive, and the absence of dedicated funding limits effective follow-up of missed children.

A limitation of the study was that we were unable to ascertain the relationship between MR vaccination status and measles at the level of individuals due to non-availability of transactional or granular data. Again, the administrative data was not validated by coverage survey due to inadequate resources and time. However, the impact on data quality was mitigated by facility level verification and validation of service delivery registers.

## 5 Conclusion

The MR vaccination coverage in Savannah Region declined probably due pre-existing weaknesses in the immunization programme accentuated by impact of the COVID-19 pandemic. The lowered population immunity likely contributed to the 2022 measles outbreak. Efforts to restore coverage was affected by vaccine stockouts and access challenges and might have contributed to the outbreak. There is the need for pragmatic actions to catch-up and vaccinate missed children, restore coverage to pre-pandemic levels, and strengthen the immunization programme towards achieving the Immunization Agenda 2030 (IA2030) trajectory.

Non-Governmental Organizations should consider leading advocacy to mobilize and prioritize local resources for vaccine acquisition to forestall the recurrent MR vaccine stockouts. While the EPI programme takes steps to resolve the denominator challenges, the districts should focus on reaching every child by strengthening immunization service delivery at facility and community levels. To bridge the immunity gap and mitigate future outbreaks, the EPI programme should consider rescheduling the postponed SIAs, and support the region to conduct periodic intensification of routine immunization. The Regional Health Directorate should build capacity of district level staff on VPD surveillance and strengthen communication with the National Public Health Reference Laboratory to improve feedback to ensure early detection and response to measles outbreaks.

## Declaration of competing interest

The authors declare that they have no known competing financial interests or personal relationships that could have appeared to influence the work reported in this paper.

## Data Availability

Data will be made available on request.
